# 1-(2-Hydr­oxy-5-methyl­phen­yl)-3-(3-methylthiophen-2-yl)prop-2-en-1-one

**DOI:** 10.1107/S1600536810017058

**Published:** 2010-05-19

**Authors:** G. B. Thippeswamy, D. Vijay Kumar, B. S. Jayashree, M. A. Sridhar, J. Shashidhara Prasad

**Affiliations:** a Department of Studies in Physics, Manasagangotri, University of Mysore, Mysore 570 006, India; b Department of Pharmaceutical Chemistry, Manipal college of Pharmaceutical Sciences, Manipal 576 104, India

## Abstract

In the structure of the title compound, C_15_H_14_O_2_S, the benzene ring is nearly coplanar with the thio­phene ring. The hydroxy group substituted at C2 position is in an antiperi­planar conformation with respect to the phenyl ring. The crystal structure exhibits weak intramolecular O—H⋯O hydrogen bonding.

## Related literature

For the bioactivity of related compounds, see: Ratty (1988 ▶); Sato *et al.* (1996 ▶); Tencate *et al.* (1973 ▶); Murakami *et al.* (1992 ▶); Gerdin & Srensso (1983 ▶); Shahidi *et al.*(1988 ▶); Jayashree *et al.* (2008 ▶); Nijveldt *et al.* (2001 ▶); Varma & Kinoshita (1976 ▶). For related structures, see: Jasinski *et al.* (2009 ▶, 2010 ▶).
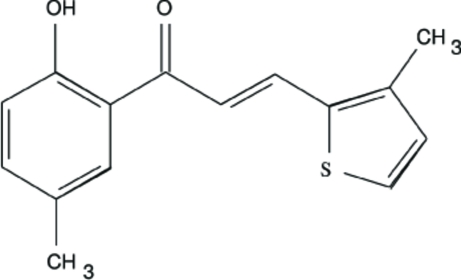


## Experimental

### 

#### Crystal data

C_15_H_14_O_2_S
*M* *_r_* = 258.33
Orthorhombic, 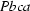

*a* = 13.6680 (12) Å
*b* = 13.3750 (8) Å
*c* = 14.5410 (14) Å
*V* = 2658.2 (4) Å^3^
*Z* = 8
Mo *K*α radiation
μ = 0.23 mm^−1^
*T* = 293 K
0.27 × 0.25 × 0.23 mm

#### Data collection

MacScience DIPLabo 32001 diffractometer
4360 measured reflections
2335 independent reflections
1742 reflections with *I* > 2σ(*I*)
*R*_int_ = 0.016

#### Refinement

*R*[*F* ^2^ > 2σ(*F* ^2^)] = 0.058
*wR*(*F* ^2^) = 0.182
*S* = 1.07
2335 reflections
166 parameters
H-atom parameters constrained
Δρ_max_ = 0.24 e Å^−3^
Δρ_min_ = −0.35 e Å^−3^

### 

Data collection: *XPRESS* (MacScience, 2002 ▶); cell refinement: *SCALEPACK* (Otwinowski & Minor, 1997 ▶); data reduction: *DENZO* (Otwinowski & Minor, 1997 ▶) and *SCALEPACK*; program(s) used to solve structure: *SHELXS97* (Sheldrick, 2008 ▶); program(s) used to refine structure: *SHELXL97* (Sheldrick, 2008 ▶); molecular graphics: *PLATON* (Spek, 2009 ▶) and *ORTEPII* (Johnson, 1976 ▶); software used to prepare material for publication: *PLATON*.

## Supplementary Material

Crystal structure: contains datablocks I, global. DOI: 10.1107/S1600536810017058/jh2143sup1.cif

Structure factors: contains datablocks I. DOI: 10.1107/S1600536810017058/jh2143Isup2.hkl

Additional supplementary materials:  crystallographic information; 3D view; checkCIF report

## Figures and Tables

**Table 1:** Hydrogen-bond geometry (Å, °)

*D*—H⋯*A*	*D*—H	H⋯*A*	*D*⋯*A*	*D*—H⋯*A*
O1—H1⋯O10	0.82	1.79	2.518 (3)	147

**Fig. 1.**
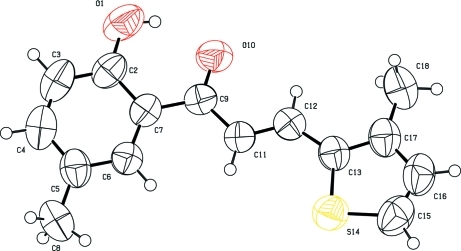
Crystal structure of the title compound with 50% probability displacement ellipsoids.

## supplementary crystallographic information

### Comment

Chalcone is the basic skeleton present in all flavonoids, which are important secondary plant metabolities reported to exhibit a wide range of biological activities such as anti-oxidant (Ratty, 1988), anti-bacterial (Sato et al., 1996), anti-fungal (Tencate et al., 1973), anti-cancer (Murakami et al., 1992), anti-HIV (Gerdin et al., 1983), anti-inflammatory (Shahidi et al., 1988) and inhibition of various enzymens such as aldose reductase, cycloxigenase, tyrosinkinase (Varma et al., 1976). The hydroxy group has exhibited a broad range of biological activities displaying anti-oxidant, anti-bacterials, anti-diabetic  properties (Jayashree et al., 2008). The unsaturated ketone group present in chalcones is believed to be responsible for the biological activity (Nijveldt et al., 2001). In view of the importance of such compounds the title compound is synthesised and its crystal structure is reported.

The molecular structure of 1-(2-hydroxy-5-methylphenyl)-3-(3-methylthiophen-2-yl)prop-2-en-1-one, consists of a phenyl ring and a thiophen ring attached to a propanone chain at 1,3-position. The bond lengths C7–C9, C9–O10, C9–C11, C11–C12, C12–C13 and bond angles C7–C9–O10, O10–C9–C11 are in good agreement with those of the similar compounds reported earlier (Jasinski et al., 2009; Jasinski et al., 2010). The torsion angle for C12-C13-C17-C18 is -1.71° and adopts -syn-periplanar conformation. The structure exhibits weak intramolecular hydrogen bond of the type O–H···O.

### Experimental

The title compound 1-(2-hydroxy-5-methylphenyl)-3-(3-methylthiophen-2-yl)prop-2-en-1-one was synthesized by dissolving 5 m mole of 5-methyl-2 hydroxyacetophenone in 15 ml of methanol taken in a conical flask, towhich 5 ml of aqueous solution of sodium hydroxide was added with stirring at room temperature. Later 5 m mole of 5-methyl-2-thiophene-carboxaldehyde was added slowly and the stirring continued for 48 hours. The mixture was then poured into ice cold water and acidified with dilute hydrochloric acid. The title compound separates as a precipitate which was collected by filtration, dried and recrystallised from methanol.

### Crystal data

**Table:**

C_15_H_14_O_2_S	*F*(000) = 1088
*M**_r_* = 258.33	*D*_x_ = 1.291 Mg m^−^^3^
Orthorhombic, *P**b**c**a*	Mo *K*α radiation, λ = 0.71073 Å
Hall symbol: -P 2ac 2ab	Cell parameters from 4360 reflections
*a* = 13.6680 (12) Å	θ = 3.2–25.0°
*b* = 13.3750 (8) Å	µ = 0.23 mm^−^^1^
*c* = 14.5410 (14) Å	*T* = 293 K
*V* = 2658.2 (4) Å^3^	Rectangular, orange
*Z* = 8	0.27 × 0.25 × 0.23 mm

### Data collection

**Table:**

MacScience DIPLabo 32001 diffractometer	1742 reflections with *I* > 2σ(*I*)
Radiation source: fine-focus sealed tube	*R*_int_ = 0.016
graphite	θ_max_ = 25.0°, θ_min_ = 3.2°
Detector resolution: 10.0 pixels mm^-1^	*h* = −15→16
ω scans	*k* = −15→15
4360 measured reflections	*l* = −17→17
2335 independent reflections	

### Refinement

**Table:**

Refinement on *F*^2^	Secondary atom site location: difference Fourier map
Least-squares matrix: full	Hydrogen site location: inferred from neighbouring sites
*R*[*F*^2^ > 2σ(*F*^2^)] = 0.058	H-atom parameters constrained
*wR*(*F*^2^) = 0.182	*w* = 1/[σ^2^(*F*_o_^2^) + (0.1131*P*)^2^ + 0.2932*P*] where *P* = (*F*_o_^2^ + 2*F*_c_^2^)/3
*S* = 1.07	(Δ/σ)_max_ < 0.001
2335 reflections	Δρ_max_ = 0.24 e Å^−^^3^
166 parameters	Δρ_min_ = −0.35 e Å^−^^3^
0 restraints	Extinction correction: *SHELXL97* (Sheldrick, 2008), Fc^*^=kFc[1+0.001xFc^2^λ^3^/sin(2θ)]^-1/4^
Primary atom site location: structure-invariant direct methods	Extinction coefficient: 0.009975

### Special details

**Table:**

Geometry. Bond distances, angles *etc*. have been calculated using the rounded fractional coordinates. All su's are estimated from the variances of the (full) variance-covariance matrix. The cell e.s.d.'s are taken into account in the estimation of distances, angles and torsion angles
Refinement. Refinement of *F*^2^ against ALL reflections. The weighted *R*-factor *wR* and goodness of fit *S* are based on *F*^2^, conventional *R*-factors *R* are based on *F*, with *F* set to zero for negative *F*^2^. The threshold expression of *F*^2^ > σ(*F*^2^) is used only for calculating *R*-factors(gt) *etc*. and is not relevant to the choice of reflections for refinement. *R*-factors based on *F*^2^ are statistically about twice as large as those based on *F*, and *R*- factors based on ALL data will be even larger.

### Fractional atomic coordinates and isotropic or equivalent isotropic displacement parameters (Å^2^)

**Table:**

	*x*	*y*	*z*	*U*_iso_*/*U*_eq_	
S14	0.03308 (6)	0.63213 (6)	0.34076 (5)	0.0917 (3)	
O1	−0.30633 (16)	0.21283 (14)	0.47357 (17)	0.1086 (9)	
O10	−0.14602 (14)	0.28250 (13)	0.41732 (14)	0.0902 (7)	
C2	−0.32536 (19)	0.3053 (2)	0.5061 (2)	0.0824 (9)	
C3	−0.4133 (2)	0.3227 (3)	0.5511 (2)	0.0998 (11)	
C4	−0.4358 (2)	0.4136 (3)	0.5850 (2)	0.0968 (13)	
C5	−0.37207 (18)	0.4947 (2)	0.57621 (16)	0.0817 (9)	
C6	−0.28521 (17)	0.47858 (19)	0.53026 (16)	0.0714 (8)	
C7	−0.25907 (16)	0.38552 (17)	0.49421 (16)	0.0681 (8)	
C8	−0.3971 (2)	0.5955 (3)	0.6154 (2)	0.1067 (14)	
C9	−0.16789 (18)	0.36901 (17)	0.44277 (16)	0.0692 (8)	
C11	−0.10438 (16)	0.45228 (18)	0.41721 (15)	0.0685 (8)	
C12	−0.02561 (16)	0.43481 (19)	0.36459 (17)	0.0702 (8)	
C13	0.04379 (16)	0.5047 (2)	0.32810 (16)	0.0724 (8)	
C15	0.1358 (3)	0.6508 (3)	0.2772 (2)	0.1040 (12)	
C16	0.1769 (2)	0.5658 (3)	0.2486 (2)	0.0996 (13)	
C17	0.12548 (19)	0.4798 (2)	0.27636 (17)	0.0830 (9)	
C18	0.1554 (2)	0.3751 (3)	0.2526 (2)	0.1080 (14)	
H1	−0.25310	0.21280	0.44750	0.1630*	
H3	−0.45760	0.27050	0.55800	0.1200*	
H4	−0.49520	0.42260	0.61500	0.1160*	
H6	−0.24220	0.53190	0.52300	0.0860*	
H8A	−0.35680	0.64560	0.58720	0.1600*	
H8B	−0.38590	0.59510	0.68060	0.1600*	
H8C	−0.46470	0.61010	0.60350	0.1600*	
H11	−0.11840	0.51680	0.43720	0.0820*	
H12	−0.01450	0.36810	0.34970	0.0840*	
H15	0.16080	0.71380	0.26380	0.1250*	
H16	0.23380	0.56360	0.21360	0.1200*	
H18A	0.14980	0.33340	0.30610	0.1620*	
H18B	0.22200	0.37500	0.23160	0.1620*	
H18C	0.11370	0.34980	0.20490	0.1620*	

### Atomic displacement parameters (Å^2^)

**Table:**

	*U*^11^	*U*^22^	*U*^33^	*U*^12^	*U*^13^	*U*^23^
S14	0.1046 (6)	0.0844 (6)	0.0861 (6)	−0.0070 (3)	0.0065 (4)	0.0020 (3)
O1	0.0995 (14)	0.0704 (12)	0.156 (2)	−0.0136 (9)	−0.0070 (13)	0.0142 (12)
O10	0.0902 (12)	0.0694 (11)	0.1110 (14)	0.0007 (9)	0.0016 (10)	−0.0092 (9)
C2	0.0757 (14)	0.0763 (16)	0.0953 (18)	−0.0082 (12)	−0.0099 (13)	0.0177 (14)
C3	0.0765 (17)	0.104 (2)	0.119 (2)	−0.0175 (16)	−0.0017 (16)	0.0356 (19)
C4	0.0715 (16)	0.125 (3)	0.094 (2)	−0.0003 (16)	0.0098 (14)	0.0259 (19)
C5	0.0724 (14)	0.106 (2)	0.0666 (14)	0.0062 (13)	0.0035 (11)	0.0083 (13)
C6	0.0704 (13)	0.0763 (15)	0.0676 (13)	−0.0012 (11)	−0.0008 (10)	0.0029 (11)
C7	0.0659 (13)	0.0703 (14)	0.0681 (13)	−0.0029 (10)	−0.0056 (10)	0.0109 (11)
C8	0.103 (2)	0.124 (3)	0.093 (2)	0.0186 (18)	0.0217 (17)	−0.0114 (19)
C9	0.0698 (13)	0.0652 (14)	0.0725 (14)	0.0004 (10)	−0.0066 (11)	0.0009 (11)
C11	0.0684 (13)	0.0682 (14)	0.0688 (13)	−0.0007 (10)	0.0007 (10)	−0.0006 (11)
C12	0.0714 (14)	0.0743 (15)	0.0650 (13)	0.0034 (10)	−0.0034 (11)	0.0013 (11)
C13	0.0704 (13)	0.0843 (17)	0.0624 (13)	0.0039 (11)	−0.0001 (10)	0.0043 (11)
C15	0.110 (2)	0.112 (2)	0.090 (2)	−0.0278 (19)	0.0034 (17)	0.0192 (18)
C16	0.0887 (19)	0.130 (3)	0.0801 (17)	−0.0134 (18)	0.0133 (14)	0.0117 (17)
C17	0.0744 (14)	0.106 (2)	0.0685 (14)	0.0040 (13)	0.0009 (11)	0.0094 (14)
C18	0.093 (2)	0.125 (3)	0.106 (2)	0.0234 (18)	0.0195 (18)	0.0011 (19)

### Geometric parameters (Å, °)

**Table:**

S14—C13	1.721 (3)	C15—C16	1.335 (5)
S14—C15	1.699 (4)	C16—C17	1.407 (5)
O1—C2	1.350 (3)	C17—C18	1.499 (5)
O10—C9	1.251 (3)	C3—H3	0.9300
O1—H1	0.8200	C4—H4	0.9300
C2—C7	1.415 (3)	C6—H6	0.9300
C2—C3	1.388 (4)	C8—H8A	0.9600
C3—C4	1.348 (5)	C8—H8B	0.9600
C4—C5	1.397 (4)	C8—H8C	0.9600
C5—C6	1.379 (3)	C11—H11	0.9300
C5—C8	1.503 (5)	C12—H12	0.9300
C6—C7	1.397 (3)	C15—H15	0.9300
C7—C9	1.470 (3)	C16—H16	0.9300
C9—C11	1.460 (3)	C18—H18A	0.9600
C11—C12	1.341 (3)	C18—H18B	0.9600
C12—C13	1.434 (3)	C18—H18C	0.9600
C13—C17	1.387 (3)		
			
C13—S14—C15	90.98 (16)	C2—C3—H3	119.00
C2—O1—H1	109.00	C4—C3—H3	119.00
O1—C2—C7	121.9 (2)	C3—C4—H4	119.00
C3—C2—C7	119.0 (3)	C5—C4—H4	119.00
O1—C2—C3	119.1 (3)	C5—C6—H6	119.00
C2—C3—C4	121.4 (3)	C7—C6—H6	119.00
C3—C4—C5	121.7 (3)	C5—C8—H8A	109.00
C4—C5—C6	117.4 (3)	C5—C8—H8B	109.00
C4—C5—C8	121.3 (2)	C5—C8—H8C	109.00
C6—C5—C8	121.3 (2)	H8A—C8—H8B	110.00
C5—C6—C7	122.8 (2)	H8A—C8—H8C	109.00
C2—C7—C6	117.8 (2)	H8B—C8—H8C	109.00
C6—C7—C9	122.8 (2)	C9—C11—H11	120.00
C2—C7—C9	119.4 (2)	C12—C11—H11	120.00
O10—C9—C11	119.2 (2)	C11—C12—H12	116.00
C7—C9—C11	121.3 (2)	C13—C12—H12	116.00
O10—C9—C7	119.5 (2)	S14—C15—H15	123.00
C9—C11—C12	119.3 (2)	C16—C15—H15	124.00
C11—C12—C13	129.0 (2)	C15—C16—H16	123.00
S14—C13—C12	123.37 (18)	C17—C16—H16	123.00
C12—C13—C17	125.2 (2)	C17—C18—H18A	110.00
S14—C13—C17	111.37 (19)	C17—C18—H18B	109.00
S14—C15—C16	113.1 (3)	C17—C18—H18C	110.00
C15—C16—C17	113.4 (3)	H18A—C18—H18B	109.00
C13—C17—C18	124.7 (2)	H18A—C18—H18C	109.00
C16—C17—C18	124.1 (2)	H18B—C18—H18C	109.00
C13—C17—C16	111.2 (2)		
			
C15—S14—C13—C12	−177.8 (2)	C2—C7—C9—C11	170.9 (2)
C15—S14—C13—C17	0.4 (2)	C6—C7—C9—C11	−7.2 (4)
C13—S14—C15—C16	−0.7 (3)	C2—C7—C9—O10	−6.3 (4)
O1—C2—C7—C6	180.0 (2)	C6—C7—C9—O10	175.6 (2)
C3—C2—C7—C6	1.1 (4)	O10—C9—C11—C12	2.1 (3)
C3—C2—C7—C9	−177.1 (2)	C7—C9—C11—C12	−175.2 (2)
O1—C2—C7—C9	1.8 (4)	C9—C11—C12—C13	177.0 (2)
O1—C2—C3—C4	179.9 (3)	C11—C12—C13—S14	−4.3 (4)
C7—C2—C3—C4	−1.3 (4)	C11—C12—C13—C17	177.8 (2)
C2—C3—C4—C5	0.3 (5)	S14—C13—C17—C16	0.0 (3)
C3—C4—C5—C8	−179.1 (3)	C12—C13—C17—C16	178.1 (2)
C3—C4—C5—C6	0.8 (4)	C12—C13—C17—C18	−1.7 (4)
C8—C5—C6—C7	179.0 (2)	S14—C13—C17—C18	−179.8 (2)
C4—C5—C6—C7	−0.9 (4)	S14—C15—C16—C17	0.8 (4)
C5—C6—C7—C2	−0.1 (4)	C15—C16—C17—C13	−0.5 (4)
C5—C6—C7—C9	178.1 (2)	C15—C16—C17—C18	179.4 (3)

### Hydrogen-bond geometry (Å, °)

**Table:**

*D*—H···*A*	*D*—H	H···*A*	*D*···*A*	*D*—H···*A*
O1—H1···O10	0.82	1.79	2.518 (3)	147
